# Empfohlene Diagnostik bei Pruritus auf primär unveränderter Haut

**DOI:** 10.1007/s00105-024-05380-1

**Published:** 2024-06-28

**Authors:** M. M. Düll, A. E. Kremer

**Affiliations:** 1 Medizinische Klinik 1, Gastroenterologie, Pneumologie, Endokrinologie, Universitätsklinikum Erlangen, Friedrich-Alexander-Universität Erlangen-Nürnberg, Erlangen, Deutschland; 2 Klinik für Gastroenterologie und Hepatologie, UniversitätsSpital Zürich, Universität Zürich, Rämistr. 100, 8091 Zürich, Schweiz

**Keywords:** Juckreiz, Leber, Myeloproliferative Neoplasien, Stufendiagnostik, Basisdiagnostik, Itch, Liver, Myeloproliferative neoplasms, Stepwise diagnostic strategy, Basic diagnostic workup

## Abstract

**Hintergrund:**

Chronischer Pruritus auf primär nichtläsionaler Haut (CPNL) stellt ein häufig auftretendes Symptom bei zahlreichen Erkrankungen aus verschiedenen medizinischen Fachgebieten dar. Die Vielzahl möglicher ätiologischer Ursachen erschwert die Diagnosestellung der zugrunde liegenden Erkrankung oft erheblich.

**Ziel der Arbeit:**

Dieser Übersichtsartikel gibt einen Überblick über die klinische, laborchemische und bildgebende Diagnostik bei CPNL.

**Material und Methoden:**

Es erfolgte eine ausführliche PubMed-Recherche zur Diagnostik bei chronischem Pruritus mit der Verwendung der Schlüsselwörter „chronic pruritus AND non-lesional skin“, „chronic itch AND non-lesional skin“, „chronic pruritus AND diagnostics“, „chronic itch AND diagnostics“, „CKD-aP“, „hepatic pruritus“, „cholestatic pruritus“ und „myeloproliferative neoplasms AND pruritus“.

**Ergebnisse:**

Zur Abklärung des CPNL wird eine Stufendiagnostik empfohlen, die sich an der Prävalenz mit Pruritus assoziierten Erkrankungen orientiert. Eine Basisdiagnostik ermöglicht eine kosteneffiziente und gezielte Evaluation beim medizinischen Erstkontakt. Die hier erhaltenen Informationen über zugrunde liegende Erkrankungen können durch spezialisierte Diagnoseverfahren noch präziser aufgearbeitet werden.

**Diskussion:**

CPNL stellt eine diagnostische Herausforderung dar. Ein schrittweises Diagnoseverfahren erleichtert, die zugrunde liegende Ätiologie zu identifizieren. Dies ist entscheidend, um Erkrankungen zu erkennen und den Pruritus gezielt mit krankheitsspezifischen Therapien zu behandeln.

## Abstract (en)

**Background:**

Chronic pruritus affecting primary non-lesional skin (CPNL) manifests as a common symptom across a spectrum of diseases spanning various medical specialties. Given the diverse etiological factors involved, diagnosing the underlying condition poses a significant challenge.

**Objectives:**

To provide a comprehensive overview of clinical, laboratory, and imaging diagnostics for CPNL.

**Materials and methods:**

A thorough literature search on the diagnostics of chronic pruritus was conducted using PubMed with specific keywords “chronic pruritus AND non-lesional skin”, “chronic itch AND non-lesional skin”, “chronic pruritus AND diagnostics”, “chronic itch AND diagnostics”, “CKD-aP”, “hepatic pruritus”, “cholestatic pruritus”, and “myeloproliferative neoplasms AND pruritus”.

**Results:**

A systematic diagnostic approach is recommended for patients with CPNL, guided by the prevalence of pruritus-associated diseases. Initial basic diagnostics facilitate a cost-effective and focused evaluation during the initial medical assessment. Information pertaining to underlying diseases can be further refined through specialized diagnostic procedures.

**Conclusions:**

CPNL often presents a diagnostic dilemma. Adopting a stepwise diagnostic strategy facilitates the identification of underlying etiologies, which is crucial for recognizing diseases and administering pruritus-specific pharmacotherapy.

Obwohl Pruritus häufiger bei klassischen dermatologischen Erkrankungen mit sichtbaren Hautveränderungen auftritt, leiden auch zahlreiche Patienten an Pruritus auf primär unveränderter, nichtläsionaler Haut. Die fehlenden primären Hautveränderungen erschweren die ätiologische Zuordnung und die erforderliche Diagnostik. Dieser Beitrag bietet einen Überblick über die gezielte klinische, laborchemische und bildgebende Diagnostik bei Pruritus auf primär unveränderter Haut sowie über die zugrunde liegenden Erkrankungen.

Pruritus übt physiologisch eine sinnvolle Warnfunktion aus, um die menschliche Haut gegenüber schädlicher Exposition wie Insekten oder Pflanzen zu schützen. Bei zahlreichen dermatologischen oder systemischen Erkrankungen entwickelt sich Pruritus über mehr als 6 Wochen jedoch zu einem chronischen Symptom. Bis zu ein Fünftel der deutschen Bevölkerung litt schon einmal im Leben an chronischem Pruritus (CP) [13]. Die Punktprävalenz von CP liegt in verschiedenen Studien aus Deutschland mindestens über 10 % [13, 18]. Trotz dieser Häufigkeit wird CP sowohl von Patienten als auch medizinischem Personal teilweise nicht ausreichend ernst genommen. Dabei bestätigen Daten aus dem Jahr 2010, dass fast die Hälfte von Patienten mit CP diesbezüglich keine medizinische Hilfe in Anspruch nimmt [18]. CP kann mild und sporadisch auftreten, aber auch moderate bis schwere Verlaufsformen annehmen. Patienten können hierdurch eine stark eingeschränkte gesundheitsbezogene Lebensqualität erleben. CP kann Schlafmangel, Tagesmüdigkeit und deutlich verringerte Leistungsfähigkeit verursachen. Es bestehen Assoziationen von CP zu Depressionen und Suizidalität.

CP tritt in einem relevanten Anteil der Fälle auch auf primär nichtläsionaler, also unveränderter Haut (CPNL) auf [20]. Pruritus kann in diesen Fällen Symptom einer systemischen, neurologischen, psychiatrischen oder malignen Erkrankung sein, die selbst einer Behandlung bedarf und nicht übersehen werden sollte [1]. Aufgrund der vielzähligen Ursachen gibt dieses Manuskript einen Überblick über CPNL und zeigt eine sinnvolle Diagnostik für die Praxis auf.

## Ätiologie

CP kann aufgrund verschiedener Aspekte eingeteilt werden. Hierzu zählt u. a. der klinische Phänotyp, insbesondere Läsionen der Haut, oder die zugrunde liegende Erkrankung, als deren Symptom CP auftritt. Die aktuelle deutsche S2k-Leitlinie „Diagnostik und Therapie des chronischen Pruritus“ fasst sämtliche Handlungsempfehlungen zu CP übersichtlich zusammen [20]. Diese Leitlinie empfiehlt die primäre Einordnung von CP anhand des klinischen Phänotyps entsprechend der internationalen Klassifikation des International Forum for the Study of Itch (IFSI) ([19]; Tab. 1). Sekundär sollte weiter nach kausalen Erkrankungsentitäten unterschieden werden. Bezüglich des CP auf primär nichtläsionaler (unveränderter) Haut (CPNL) sind diverse auslösende u. a. dermatologische, (hämato)onkologische, gastroenterologisch/hepatologische, nephrologische, neurologische, psychologische/psychiatrische, infektiologische und medikamentöse Differenzialdiagnosen in Erwägung zu ziehen, die in Tab. 2 mit Beispielen zusammengefasst sind. Die Primär- und Sekundärcharakterisierung mittels Subkategorien trägt der Komplexität von Patienten mit CP Rechnung [25].

**Tab. 1:** Klassifikation des chronischen Pruritus. (Adaptiert von [19, 20])

Klinischer Phänotyp	Bezeichnung
Pruritus bei gleichzeitigem Vorliegen von Hautveränderungen, zumeist im Rahmen einer Hauterkrankung (Früher auch: Pruritus cum materia)	IFSI I: chronischer Pruritus auf primär läsionaler (veränderter) Haut (CPL)
Pruritus ohne initiales Vorliegen von Hautveränderungen (Früher auch: Pruritus sine materia)	IFSI II: chronischer Pruritus auf primär nichtläsionaler (unveränderter) Haut (CPNL)
Vorherrschen von chronischen Kratzläsionen (z. B. chronische Prurigo, Lichen simplex), die eine Einteilung in die erste oder zweite Gruppe nicht möglich machen	IFSI III: chronischer Pruritus mit schweren Kratzläsionen

*IFSI* International Forum for the Study of Itch

**Tab. 2:** Differenzialdiagnostische Kategorien bei CPNL (chronischer Pruritus auf unveränderter Haut). (Adaptiert von [20, 24])

Differenzialdiagnostische Kategorien	Beispiele
Dermatologische Erkrankungen („Unsichtbare Dermatosen“)	Asteatotisches Ekzem, beginnendes bullöses Pemphigoid, Porphyrien, Dermatitis herpetiformis Duhring, polymorphe Lichtdermatose, kutane Mastozytose
Hämatologische und myeloproliferative Erkrankungen	Myeloproliferative Neoplasien (z. B. Polycythaemia vera, essenzielle Thrombozytose), Lymphome, Plasmozytom, Hypereosinophiliesyndrome, systemische Mastozytose, Eisenmangel
Solide Tumoren	Tumoren der Mamma, Lunge, des Magens, Pankreas, Kolons/Rektums, der Prostata, Zervix, Karzinoide und Sarkome
Metabolische und endokrine Erkrankungen	Diabetes mellitus, Hypo‑/Hyperthyreose, Hyperparathyreoidismus, (Peri‑)Menopause
Hepatologische Erkrankungen	Primär biliäre Cholangitis, primär/sekundär sklerosierende Cholangitis, IgG4-assoziierte Cholangiopathie, Alagille-Syndrom, hereditäre Cholestasesyndrome (BRIC, PFIC), intrahepatische Schwangerschaftscholestase (ICP), Leberzirrhose (unabhängig von der ursächlichen Erkrankung), obstruktive Cholestase benigner und maligner Genese (u. a. Gallengangssteine, biliäre Karzinome, Pankreaskopfkarzinome)
Nephrologische Erkrankungen	Chronische Niereninsuffizienz jeglicher Genese (insbesondere bei Dialysepflichtigkeit)
Malassimilationssyndrome	Zöliakie, Laktoseintoleranz, Mangel an B‑/D-Vitaminen
Neurologische Erkrankungen	Multiple Sklerose, Gehirntumoren, lokalisierte Spinalkanalstenosen/Radikulopathien (brachioradialer Pruritus, Notalgia paraesthetica), Neuropathien (postherpetische Neuralgie, Kleinfaserneuropathien, Vulvodynie)
Infektiologische Krankheitsbilder	Akute/chronische HBV-/HCV-/HIV-/HSV-Infektionen, Helicobacter-pylori-Infektion, Parasitosen
Psychiatrische und psychosomatische Erkrankungen	Anorexia nervosa, Schizophrenie, Depression, Angststörungen, somatoformer Pruritus, taktile Halluzinosen
Medikamentenassoziierte Nebenwirkung	U. a. Hydroxyethylstärke(HES)-assoziierter Pruritus; diverse weitere Medikamente s. Tab. 9 der S2k-Leitlinie Chronischer Pruritus [20]

*Ig* Immunglobulin, *BRIC* „benign recurrent intrahepatic cholestasis“, *PFIC* „progressive familial intrahepatic cholestasis“, *HBV* Hepatitis-B-Virus, *HCV* Hepatitis-C-Virus, *HIV* humanes Immundefizienzvirus, *HSV* Herpes-simplex-Virus

Bei Pruritus auf primär läsionaler Haut (CPL) liegen in den meisten Fällen dermatologische Erkrankungen zugrunde, wie z. B. entzündliche Dermatosen, infektiöse Dermatosen, Autoimmundermatosen, Genodermatosen, Schwangerschaftsdermatosen sowie Neoplasien [20].

Im klinischen Alltag gestalten sich die Klassifikation des CP als Symptom einer zugrunde liegenden Erkrankung und die praktische diagnostische Aufarbeitung häufig schwieriger, wenn keine Hautläsionen oder stark ausgeprägte Kratzexkoriationen (IFSI III, s. Tab. 1) vorliegen [20]. In der Pruritusdiagnostik und -therapie, insbesondere bei CPNL, ist ein interdisziplinärer Ansatz entscheidend. Dies spiegelt sich bereits in den beteiligten Autor/-innen der deutschen S2k-Leitlinie zum CP wider, die aus verschiedenen Fachbereichen der Dermatologie, Nephrologie, Gastroenterologie/Hepatologie, Versorgungsforschung und medizinischen Psychologie stammen [20].

In der Pruritusdiagnostik und -therapie ist ein interdisziplinärer Ansatz entscheidend

Primäre Ansprechpartner für CP sind in der Regel aus dem hausärztlichen oder niedergelassenen dermatologischen Fachbereich [11]. Daher ist es hilfreich, mit einer initialen Basisdiagnostik zu starten, die bei unauffälligen Befunden ausgebaut werden kann.

Häufiger besteht die Sorge einer malignen Grunderkrankung bei Vorliegen von CPNL. Zwei große Kohortenstudien mit 8744 und 12.813 Patienten wiesen bei der Ursachenabklärung des CPNL erhöhte Malignomraten lediglich für hämatologische Erkrankungen, insbesondere Lymphome und myeloproliferative Neoplasien sowie Gallengangskarzinome auf [4, 9].

Trotz intensiver Abklärung kann die Ursache des CPNL bei manchen Patienten ungeklärt bleiben. Hier variiert die Zahl der betroffenen Fälle in der Literatur je nach untersuchtem Kollektiv zwischen 13 und 50 % [16].

Hierbei ist zu beachten, dass auch ein prämonitorischer Pruritus vorliegen kann, der einer Erstdiagnose der auslösenden Grunderkrankung um Monate bis Jahre vorausgeht [20]. Daher kann eine erneute Kontrolle der Basisdiagnostik nach 6 bis 12 Monaten bzw. in Abhängigkeit der klinischen Entwicklung sinnvoll sein.

## Basisdiagnostik

Aufgrund der diversen Ursachen des CPNL ist eine Stufendiagnostik empfehlenswert. Es sollte eine initiale Basisdiagnostik erfolgen, die auch kostengünstig hausärztlich oder im niedergelassenen dermatologischen Bereich durchgeführt werden kann (Tab. 3; [20]). Bei spezifischen Auffälligkeiten, wie z. B. erhöhten Transaminasen, Cholestase- oder Retentionsparametern, sollte Kontakt zur entsprechenden Fachdisziplin zur Mitbeurteilung aufgenommen werden.

**Tab. 3:** Basisdiagnostik (Anamnese und Labordiagnostik) des chronischen Pruritus auf primär nichtläsionaler Haut (CPNL). (Adaptiert von [20])

Basisdiagnostisches Vorgehen	
**Anamnese**	
*Pruritus*	Beginn, Dauer
	Intensität
	Lokalisation
	Qualität („Jucken wie Insektenstich“, Brennen, neuropathische Empfindungen etc.)
	Verlauf: Abhängigkeit von Tages- oder Jahreszeit, kontinuierlich vs. attackenartig
	Faktoren mit Verbesserung (z. B. Kälte)/Verschlechterung Pruritus (z. B. Wasserkontakt)
	Auftreten von Pruritus im engeren Umfeld
	Kratzverhalten
	Zeitlicher Zusammenhang zu bestimmten Ereignissen (Operationen, Medikamenteneinnahmen, neuen Erkrankungen etc.)
	Effektive/nichteffektive Vortherapien
	Patiententheorie zur Genese des Pruritus
	Psychosoziale Faktoren
	Einschränkung der Lebensqualität, Leidensdruck, Schlafstörungen
*Erweiterte Anamnese*	Vorerkrankungen
	Medikamente
	Operationen
	Allergien (Typ I, Typ IV)
	Atopische Diathese
	B‑Symptomatik
	Reiseanamnese
	Schwangerschaft
**Labordiagnostik**	
Blutsenkungsgeschwindigkeit (BSG) und C‑reaktives Protein (CRP)	
Differenzialblutbild, Ferritin	
Bilirubin, Transaminasen (GPT [ALAT], GOT [ASAT]), Gammaglutamyl-Transferase (GGT), alkalische Phosphatase	
Kreatinin, Harnstoff, geschätzte glomeruläre Filtrationsrate (eGFR), K^+^, Urin (Streifentest)	
Nüchternglukose	
Laktatdehydrogenase (LDH)	
Thyroidea-stimulierendes Hormon (TSH)	

*GPT* Glutamat-Pyruvat-Transaminase, *ALAT* Alanin-Aminotransferase, *GOT* Glutamat-Oxalacetat-Transaminase, *ASAT* Aspartat-Aminotransferase, *K*^*+*^ Kaliumionen

**Abb. 1:**
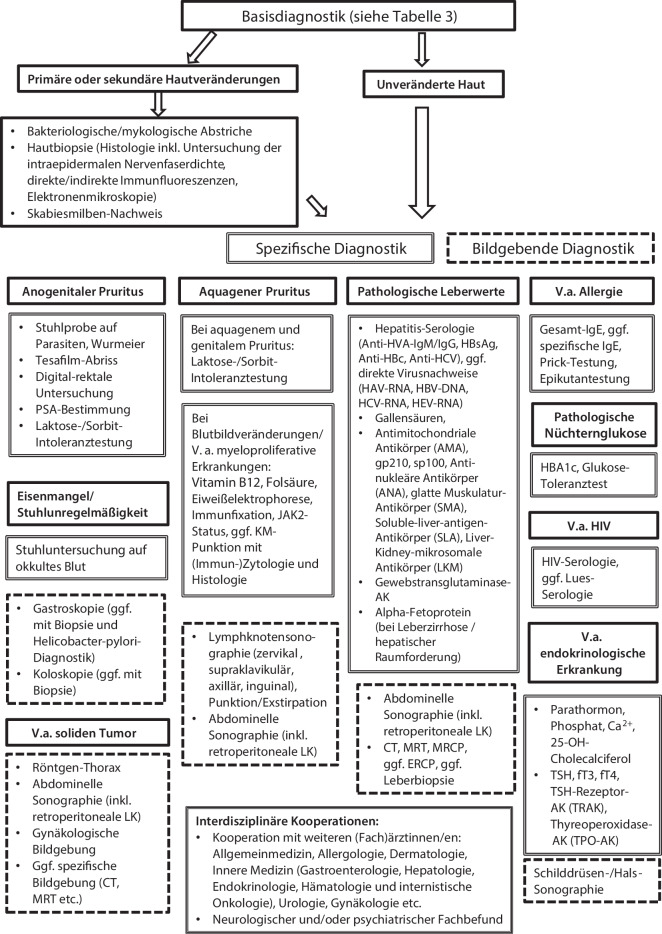
Spezifische Diagnostikoptionen bei chronischem Pruritus auf primär nichtläsionaler Haut (CPNL). *V.a.* Verdacht auf, *PSA* prostataspezifisches Antigen, *LK* Lymphknoten, *CT* Computertomographie, *MRT* Magnetresonanztomographie, *KM* Knochenmark, *Anti-HVA-IGM* Antikörper gegen Hepatitis-A-Virus vom Typ Immunglobulin M, *Ig* Immunglobulin, *HBsAg* „Hepatitis B surface antigen“, *Anti-HBc* Antikörper gegen Hepatitis-B-Core-Antigen, *Anti-HCV* Hepatitis-C-Antikörper, *HAV* Hepatitis-A-Virus, *RNA* Ribonukleinsäure, *HBV* Hepatitis-B-Virus, *DNA* Desoxyribonukleinsäure, *HCV* Hepatitis-C-Virus, *HEV* Hepatitis-E-Virus, *MRCP* Magnetresonanz-Cholangiopankreatikographie, *ERCP* endoskopisch retrograde Cholangiopankreatikographie, *HIV* humanes Immundefizienzvirus, *CA*^*2+*^ Kalzium, *TSH* Thyroidea-stimulierendes Hormon, *fT3* freies Trijodthyronin, *fT4* freies Tetrajodthyronin. (Adaptiert nach Tab. 8 der S2k-Leitlinie „Diagnostik und Therapie des chronischen Pruritus“ [20])

Bei nicht ausreichend wegweisenden Befunden in den Basisanalysen werden weiterführende Untersuchungen empfohlen, die sich an den häufigen mit CPNL-assoziierten Erkrankungen aus allen Fachbereichen orientieren (Tab. 3; Abb. 1). Die initiale Diagnostik beinhaltet neben einer Laboranalyse auch eine gezielte Anamneseerhebung und körperliche Untersuchung.

In der Anamnese sollten die Charakteristika des CP abgefragt werden. Hierzu zählen die Eigenschaften des Pruritus und die allgemeine Erkrankungsanamnese u. a. Vorerkrankungen, Medikamente und Allergien (s. Tab. 3). Da Juckempfindung subjektiv ist, erfolgt die Einschätzung durch Patientenbefragung [20]. Zur ersten Beurteilung wird die Pruritusintensität in der klinischen Praxis über eine numerische Ratingskala erhoben [17, 20]. Dabei wird entweder die durchschnittliche oder höchste Intensität der letzten 24 h abgefragt, da längere Erinnerungszeiträume von Patienten unzuverlässig eingeschätzt werden. Weitere Details des Pruritus werden im Anamnesegespräch oder über strukturierte Fragebögen erhoben. Seit 2011 ist hierzu in Deutschland ein Pruritus-Fragebogen der Arbeitsgemeinschaft Pruritusforschung (AGP) verfügbar [23]. Um den zeitlichen Verlauf des CP besser zu überwachen, können auch Tagebücher in analoger oder digitaler Form (z. B. kostenfreie ItchyApp) zum Einsatz kommen [20].

Die psychischen Auswirkungen des Pruritus sowie psychische Komorbiditäten und deren ggf. bestehende Therapien sollten im Rahmen der Anamnese erfragt werden, und ggf. sollte eine weitere spezialisierte Diagnostik bzw. Behandlung angeschlossen werden (z. B. psychosomatische Grundversorgung, Schulungsprogramme, psychosomatisch/psychiatrisch fachärztliche Diagnostik/Behandlung, Psychotherapie). In manchen Fällen liegen neben organischen Pruritusursachen auch relevante psychische und/oder psychosomatische Faktoren vor, die einer entsprechenden Mitbehandlung bedürfen. Derartige Faktoren können nach Ausschluss organischer Ursachen auch überwiegend oder alleinig für CP verantwortlich sein, der als „somatoformer Pruritus“ klassifiziert wird [14]. Insgesamt sind in der Diagnostik des CP Screeningfragen bezüglich Angst- und Depressionsstörungen sinnvoll [20]. Pruritus, der im Rahmen einer Zönästhesie oder schizophrenen Psychose auftritt, sollte als Symptom einer Schizophrenie klassifiziert werden und bedarf psychiatrischer Therapie der Grunderkrankung.

In der Anamnese sollte die Erfassung des individuellen Kratzverhaltens erfolgen. Bei CPNL mit ausgeprägten Kratzläsionen (ISFI III) kann die Unterscheidung zwischen primären (meist dermatologische Grunderkrankungen) und sekundären Hautveränderungen (durch intensives Kratzen bedingt) herausfordernd sein. In der körperlichen Untersuchung kann eine Aussparung des mittleren Rückens als sog. „Schmetterlingszeichen“ beobachtet werden. Dies spricht für sekundär bedingte Hautveränderungen, die an gut zugänglichen Hautpartien entstanden sind und initial ein CPNL vorlag. Die diagnostische Evaluation soll dann als Pruritus auf primär nichtläsionaler Haut erfolgen.

Auch die Lokalisation des Pruritus kann zumindest grundlegende Hinweise auf eine mögliche Genese geben. Pruritus bei Nierenerkrankungen (CKD-assoziierter Pruritus [CKD-aP]) tritt typischerweise an Kopf, Rücken oder Shunt-Arm auf, wenngleich 25–50 % der Patienten über einen generalisierten Pruritus berichten [15]. Hepatischer Pruritus ist häufiger an den Extremitäten lokalisiert, charakteristisch sind Handinnenflächen und Fußsohlen betroffen, jedoch generalisiert der Pruritus im Krankheitsverlauf ebenfalls häufig [2]. Insbesondere Patienten mit myeloproliferativen Neoplasien können unter einem aquagenen Pruritus leiden mit stechendem Jucken nach Wasserkontakt bzw. nach dem Abtrocknen der Haut. In einer Studie bei Patienten mit aquagenem Pruritus wurde in 25 % der Fälle eine Laktoseintoleranz als möglicher Auslöser identifiziert [7]. Bei anogenitalem Pruritus sollten parasitäre Erkrankungen (insbesondere Wurmbefall), Hämorrhoidalleiden, angewandte Kosmetika, übertriebene Hygienemaßnahmen, die gynäkologische und urologische Vorgeschichte und sexuelle Besonderheiten in die Diagnostik miteinbezogen werden [22].

Bestimmte Formen des neuropathischen Pruritus treten an spezifischen Lokalisationen auf und können zusätzlich mit Dys‑/Parästhesien einhergehen. So betrifft brachioradialer Pruritus typischerweise den dorsolateralen Unterarm und Notalgia paraesthetica den Bereich zwischen den Schulterblättern. Pruritus im Rahmen von Kleinfaserneuropathien wie im Rahmen eines Diabetes mellitus ist klassischerweise „strumpf-/handschuhförmig“ an den Extremitäten zu erwarten.

Die Lokalisation des Pruritus kann grundlegende Hinweise auf eine mögliche Genese geben

Die klinische Untersuchung sollte darüber hinaus eine umfassende Inspektion der gesamten Haut sowie Schleimhäute, Kopfhaut, Haare, Nägel und Anogenitalregion beinhalten [20]. Hierzu zählt auch die Einordnung von Effloreszenzen, Hautkolorit und Hautzeichen systemischer Erkrankungen. Bei vorliegender Leberzirrhose können Pruritus-unabhängige Auffälligkeiten bestehen wie Skleren‑/Hautikterus, Spidernävi, Palmar‑/Plantarerythem, Lacklippen/Lackzunge, Caput medusae (oberflächliche Umgehungskreisläufe) oder eine Bauchglatze [3]. Zusätzlich ist eine orientierende internistische körperliche Untersuchung sinnvoll (Herz, Lunge, Leber, Milz, Nieren und Lymphknotenstationen).

**Abb. 2:**
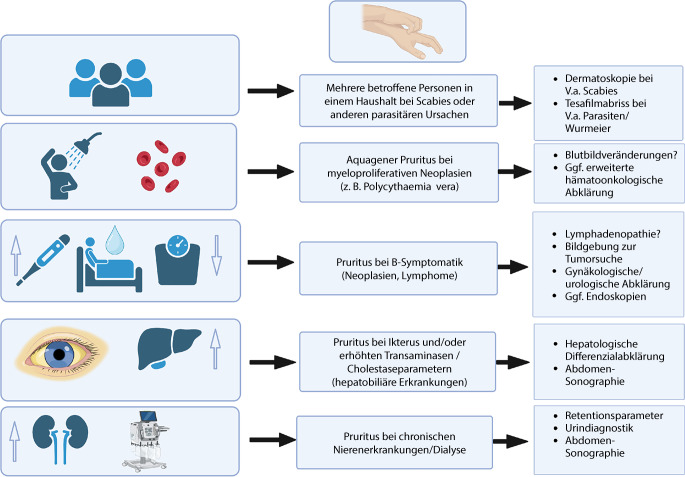
Spezifische klinische Konstellationen bei verursachenden Erkrankungen des chronischen Pruritus auf primär nichtläsionaler Haut (CPNL). *V.a.* Verdacht auf. (Adaptiert nach Tab. 5 der S2k-Leitlinie „Diagnostik und Therapie des chronischen Pruritus“ [20]. Erstellt mit BioRender.com)

Laborchemische Basisparameter sollten zur weiteren Diagnostik erhoben werden. Hierzu zählen u. a. hämatologische Werte, Leber- und Nierenwerte (inklusive Urintest) sowie Nüchternglukose, LDH (Laktatdehydrogenase) und TSH (Thyroidea-stimulierendes Hormon) (s. Tab. 3). Bereits in der Basisdiagnostik mittels Anamnese, körperlicher Untersuchung und Labordiagnostik können sich wichtige Hinweise auf bestimmte zugrunde liegende Erkrankungen ergeben (Abb. 2), und die weitere Abklärung kann zielgerichteter erfolgen.

## Spezielle Diagnostik

Finden sich in den Basisuntersuchungen Auffälligkeiten, sollten bereits gezielte weiterführende Untersuchungen angeschlossen werden, und ggf. sollte eine Überweisung an eine entsprechende medizinische Fachrichtung erfolgen. Aufgrund der Vielzahl an unterschiedlichen zugrunde liegenden Erkrankungen bei CPNL ist die weiterführende Diagnostik in Abb. 1 nochmals übersichtlich zusammengefasst.

Die zugrunde liegende Ursache des Pruritus zu identifizieren ist ein häufiger Patientenwunsch, aber auch relevant für gezielte therapeutische Maßnahmen, insbesondere da in den letzten Jahren vermehrt Medikamente zur Behandlung des Pruritus in spezifischen Erkrankungsentitäten zugelassen wurden [1], von denen betroffene Patienten profitieren können. Positiv ist hier hervorzuheben, dass Pruritus im Rahmen zahlreicher klinischer Studien als relevantes Symptom entweder als primärer Studienendpunkt oder auch als sekundärer Outcome-Parameter bei krankheitsmodifizierenden Medikamenten evaluiert wird.

Der peripher wirksame κ‑Opioid-Rezeptor(KOR)-Agonist Difelikefalin wurde 2022 als erstes Medikament zur spezifischen Behandlung des Pruritus bei Nierenerkrankungen (CKD-aP) zugelassen und ist insbesondere für Patienten unter Hämodialyse hilfreich [5]. Das Medikament wird 3‑mal/Woche intravenös nach der Dialysebehandlung verabreicht.

Es gibt eine wachsende Anzahl an Arzneimitteln zur Behandlung des Pruritus in spezifischen Erkrankungsentitäten

Bei myeloproliferativen Neoplasien können Inhibitoren des überaktivierten JAK-STAT(„Janus kinase-signal transducers and activators of transcription“)-Signalwegs wie Ruxolitinib nicht nur die Grunderkrankung therapieren, sondern auch Pruritus deutlich reduzieren [10].

Im Bereich des hepatischen Pruritus wurden für hereditäre Cholestasesyndrome kürzlich 2 IBAT(ilealer Gallensalztransporter)-Inhibitoren, Odevixibat und Maralixibat, zugelassen [6, 21]. Weitere Medikamente aus der Klasse der IBAT-Inhibitoren werden bei weiteren Gallenwegserkrankungen in klinischen Studien aktuell noch untersucht. In 2 großen Phase-III-Studien von Agonisten am Transkriptionsfaktor PPAR („peroxisome proliferator-activated receptor“) führte die Behandlung mittels Seladelpar und Elafibranor zur deutlichen laborchemischen Verbesserung bei Patienten mit primär biliärer Cholangitis (PBC). Wenngleich Elafibranor nur einen antipruriginösen Trend aufwies, erreichte Seladelpar auch den sekundären Endpunkt einer deutlichen Pruritusreduktion [8, 12].

## Fazit für die Praxis

- Bei chronischem Pruritus auf primär nichtläsionaler Haut (CPNL) sollten dermatologische, (hämato)onkologische, gastroenterologische/hepatologische, nephrologische, neurologische, psychologische/psychiatrische, infektiologische und medikamentöse Differenzialdiagnosen evaluiert werden.
- Für die initiale und schnelle Diagnostik wird eine Pruritus-spezifische Anamnese, körperliche Untersuchung und laborchemische Basisdiagnostik empfohlen.
- Anamnestische Details können bereits Hinweise auf die zugrunde liegende Erkrankung geben: So tritt hepatischer Pruritus häufiger an Handinnenflächen oder Fußsohlen auf, aquagener Pruritus kann auf myeloproliferative Neoplasien hinweisen.
- Je nach Befunden der Basisdiagnostik sollten zur weiteren Abklärung des CPNL eine spezifischere Labordiagnostik, Bildgebung und interdisziplinäre Beratung angeschlossen werden.
- Die genaue ätiologische Einordnung von CPNL ist sinnvoll, um behandlungsbedürftige Grunderkrankungen zu identifizieren, und den Pruritus individualisiert mit spezifischen Medikamenten behandeln zu können.

## Abkürzungen

AGP: Arbeitsgemeinschaft Pruritusforschung
AK: Antikörper
ALAT: Alanin-Aminotransferase
AMA: Antimitochondriale Antikörper
ANA: Antinukleäre Antikörper
Anti-HBc: Antikörper gegen Hepatitis-B-Core-Antigen
Anti-HCV: Hepatitis-C-Antikörper
Anti-HVA-IGM: Antikörper gegen Hepatitis-A-Virus vom Typ Immunglobulin M
ASAT: Aspartat-Aminotransferase
BRIC: Benign recurrent intrahepatic cholestasis
BSG: Blutsenkungsgeschwindigkeit
CA^2+^: Kalzium
CKD-aP: „Chronic kidney disease“-assoziierter Pruritus
CP: Chronischer Pruritus
CPL: Pruritus auf primär läsionaler Haut
CPNL: Chronischer Pruritus auf primär nichtläsionaler Haut
CRP: C‑reaktives Protein
CT: Computertomographie
DNA: Desoxyribonukleinsäure
eGFR: Geschätzte glomeruläre Filtrationsrate
ERCP: Endoskopisch retrograde Cholangiopankreatikographie
fT3: Freies Trijodthyronin
fT4: Freies Tetrajodthyronin
GGT: Gammaglutamyl-Transferase
GOT: Glutamat-Oxalacetat-Transaminase
GPT: Glutamat-Pyruvat-Transaminase
HAV: Hepatitis-A-Virus
HBsAg: Hepatitis B surface antigen
HBV: Hepatitis-B-Virus
HCV: Hepatitis-C-Virus
HES: Hydroxyethylstärke
HEV: Hepatitis-E-Virus
HIV: Humanes Immundefizienzvirus
HSV: Herpes-simplex-Virus
IBAT: Ilealer Gallensalztransporter
ICP: Intrahepatische Schwangerschaftscholestase
IFSI: International Forum for the Study of Itch
Ig: Immunglobulin
JAK-STAT: Janus kinase-signal transducers and activators of transcription
K^+^: Kaliumionen
KM: Knochenmark
KOR: Kappa-Opioid-Rezeptor
LDH: Laktatdehydrogenase
LK: Lymphknoten
LKM: Liver-Kidney-mikrosomale Antikörper
MRCP: Magnetresonanz-Cholangiopankreatikographie
MRT: Magnetresonanztomographie
PBC: Primär biliäre Cholangitis
PFIC: Progressive familial intrahepatic cholestasis
PPAR: Peroxisome proliferator-activated receptor
PSA: Prostataspezifisches Antigen
RNA: Ribonukleinsäure
SLA: Soluble-liver-antigen-Antikörper
SMA: Glatte Muskulatur-Antikörper
TPO-AK: Thyreoperoxidase-Antikörper
TRAK: Thyroidea-stimulierendes Hormon-Rezeptor-Antikörper
TSH: Thyroidea-stimulierendes Hormon
V.a.: Verdacht auf
